# Challenges in Diagnosing SAPHO Syndrome: A Multidisciplinary Perspective

**DOI:** 10.7759/cureus.71897

**Published:** 2024-10-19

**Authors:** Maryam Zahid, Varshini Vishwanatham, Iqra Nasir

**Affiliations:** 1 General Practice, Coldside Medical Practice, Dundee, GBR; 2 Internal Medicine, Ninewells Hospital, Dundee, GBR; 3 Internal Medicine, Islamic International Medical College, Rawalpindi, PAK

**Keywords:** acne, arthritis, arthropathy, case report, hyperostosis, osteitis, pustulosis, sapho syndrome, synovitis

## Abstract

SAPHO syndrome (synovitis, acne, pustulosis, hyperostosis, osteitis syndrome) is a systemic inflammatory disease characterized by a combination of both osteoarticular and dermatological manifestations.

We encountered an interesting case of a 54-year-old female patient who presented with symptoms of recurrent chest infections. Extensive investigations, including laboratory tests and imaging, were conducted with an initial suspicion of malignancy, but the patient was ultimately diagnosed with SAPHO. Treatment was initiated with a corticosteroid and an immunosuppressant, resulting in mild symptom improvement.

This case emphasizes the importance of considering SAPHO syndrome in patients with unexplained musculoskeletal and dermatological symptoms, as early diagnosis and treatment can significantly impact patient outcomes.

## Introduction

SAPHO syndrome (synovitis, acne, pustulosis, hyperostosis, osteitis syndrome) is a rare systemic inflammatory disease first described by Chamot et al. in 1987 [1]. A retrospective study states that the most common symptom of this condition is anterior chest wall pain (mainly involving manubriosternal and sternoclavicular joints), followed by cutaneous symptoms, peripheral arthritis, and sacroiliac pain [2]. The syndrome can occur at any age but usually appears between childhood and middle age, follows a prolonged relapsing and remitting course, and is frequently known to be misdiagnosed as commonly occurring conditions such as malignancy, infectious osteomyelitis/arthritis, or arthropathies [3,4]. Patients commonly present with anterior chest wall pain, back pain, palmoplantar pustulosis, and acne.

Diagnosis is often challenging due to the rarity and variability of the symptoms and commonly involves a fusion of clinical findings, radiological evidence, and sometimes biopsies.

## Case presentation

A 54-year-old British White woman with no significant past medical history and no remarkable family history presented to primary care with recurrent chest infections associated with drenching night sweats. She was invariably started on multiple consecutive courses of antibiotics, which had little to no effect on her gradually worsening symptoms.

She eventually came back with complaints of constant dull, aching pain and stiffness of her shoulders, ribs, and clavicles with associated difficulty in mobilization. The patient did not complain of any skin lesions bothering her. On examination, she was found to be tender on palpation over the sternum. No cutaneous manifestations, such as acne or palmoplantar pustulosis, were seen at this point. The patient's vitals were normal.

She soon underwent blood tests, which demonstrated a raised red blood cell count, normal hemoglobin, and normal C-reactive protein (CRP) (Table 1). In due course, connective tissue disorder and myeloproliferative screening were carried out, which came back negative.

**Table 1:** Laboratory Investigations

Parameters (Units)	Results	Normal Range
Haemoglobin (g/L)	162	130-180
White cell count (x10^9^/L)	8.3	4.0-11.0
Platelet (x10^9^/L)	207	150-400
Red cell count (x10^12^/L)	6.5	4.5-6.0
Haematocrit	0.454	0.40-0.52
C-Reactive Protein (mg/L)	< 0.5	0.0-10.0

Initially, a plain radiograph of the chest showed bilateral basal pleuro-parenchymal scarring with an infiltrate at the left base. Antibiotics were started, and the left basal inflammatory infiltrate resolved on a 6-week repeat chest X-ray (CXR). There was persisting fine scarring adjacent to the horizontal fissure and a longstanding right basal pleural reaction.

**Figure 1:**
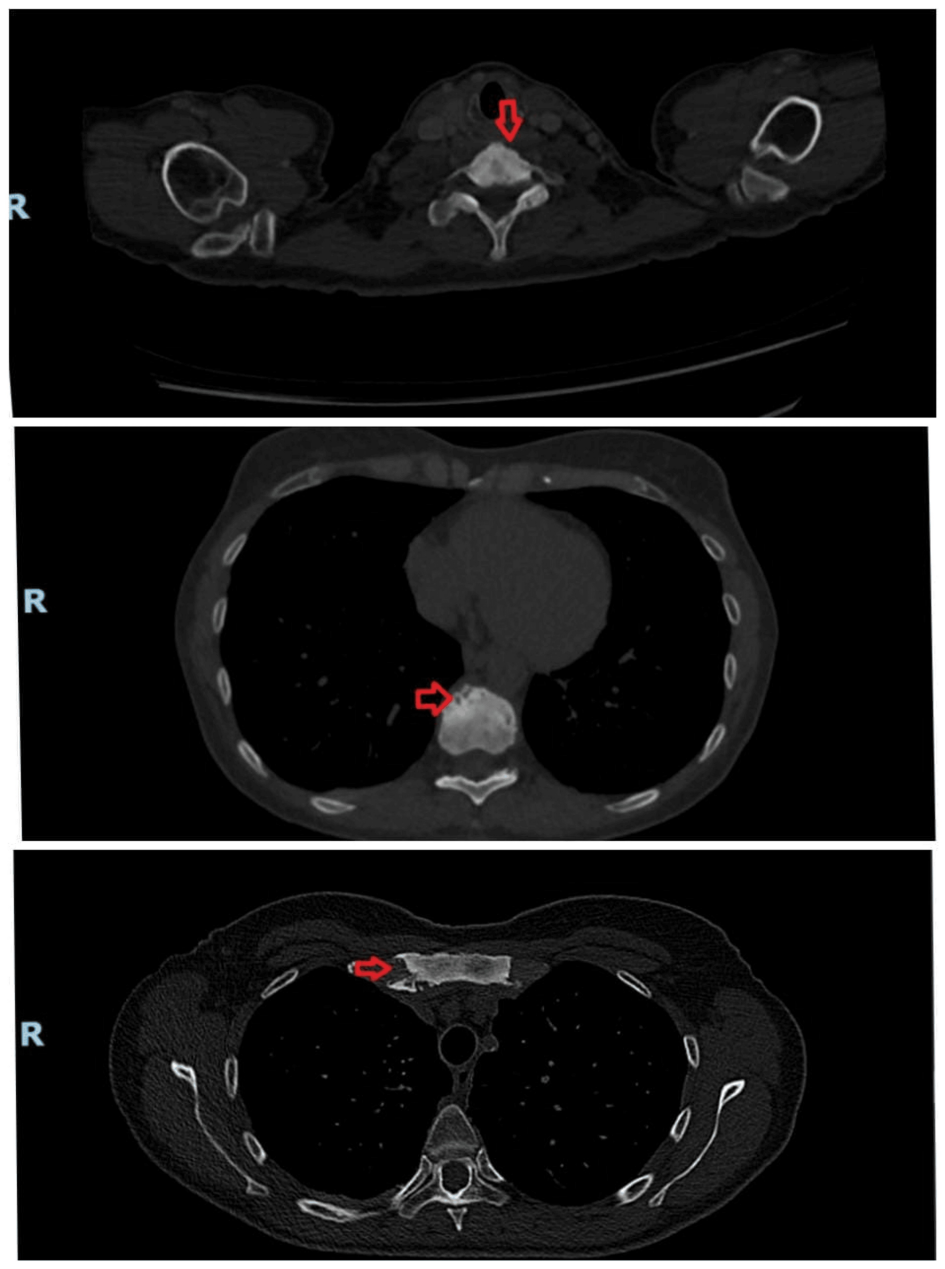
Axial CT scan in the bone window shows multiple sclerotic lesions in the vertebrae (cervical and thoracic) and in the manubrium

Before long, a CT scan of the chest was organized to investigate the cause of her persistent symptoms further. The report unveiled suspicious lung nodules with sclerotic bony deposits in the manubrium sternum and the lower cervical and mid-thoracic vertebrae, which were thought to be bony metastases from an unknown primary origin (Figure 1). The report also stated that she had bilateral dense breasts with benign fine calcifications within the glandular tissue but no definite enhancing nodules and no axillary lymphadenopathy. Breast cancer was the top differential at this stage. She was soon invited to get a triple breast assessment done, which came back negative as well. A CT of the abdomen and pelvis came back negative for malignancy, and an upper GI endoscopy was unremarkable.

Ultimately, a multidisciplinary team (including oncologists, anesthetists, and radiologists) discussion was conducted to identify the primary source of the malignancy. The decision to undergo an MRI scan was made.

**Figure 2:**
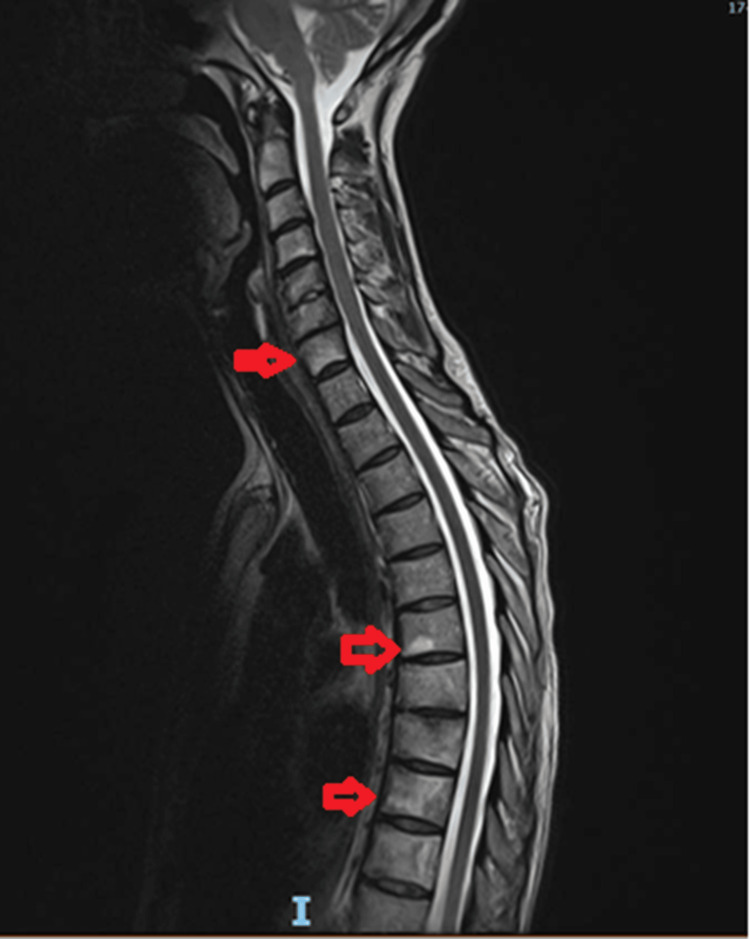
Sagittal T2W MRI image shows zones of vertebral bone marrow abnormality (sclerosis) extending from C5-C7 and T6-T10

Interestingly, while she was lying down in the MRI machine, the radiologist noticed a scaling rash on the sole of her foot and minor pustulation/scarring on the tips of her fingers. The MRI report confirmed sclerotic changes in the manubrium, cervical (C5-C7), and thoracic (T6 to T10) vertebrae (Figure 2).

The MRI report suggesting osteoarticular involvement, along with the clinical manifestations, eventually led to the diagnosis of SAPHO syndrome after a prolonged presentation of 1.5 years. The patient was started on prednisolone 15 mg daily and azathioprine 100mg daily for six months.

**Figure 3:**
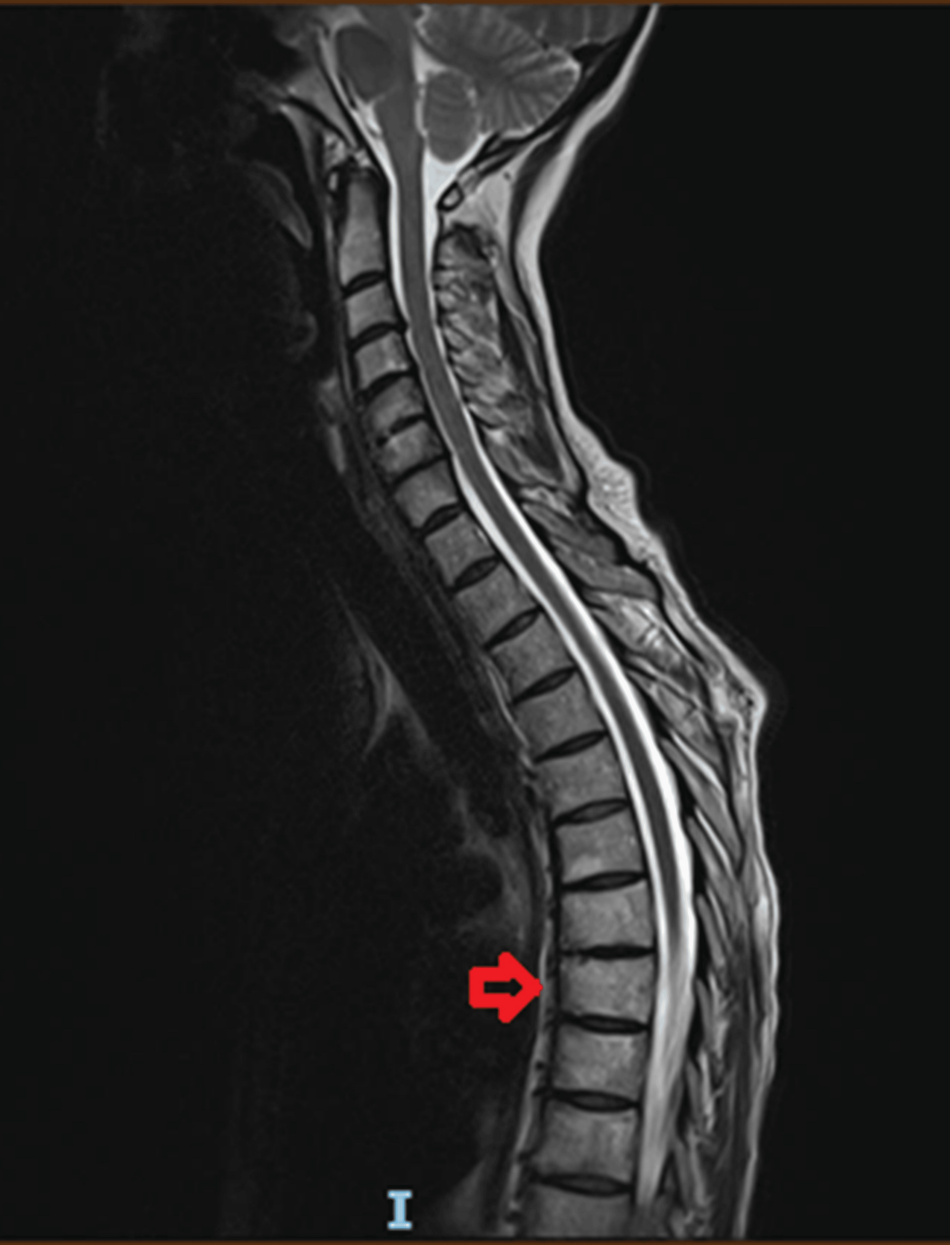
Sagittal T2W image shows some healing in the depression of the superior endplate of T8

**Figure 4:**
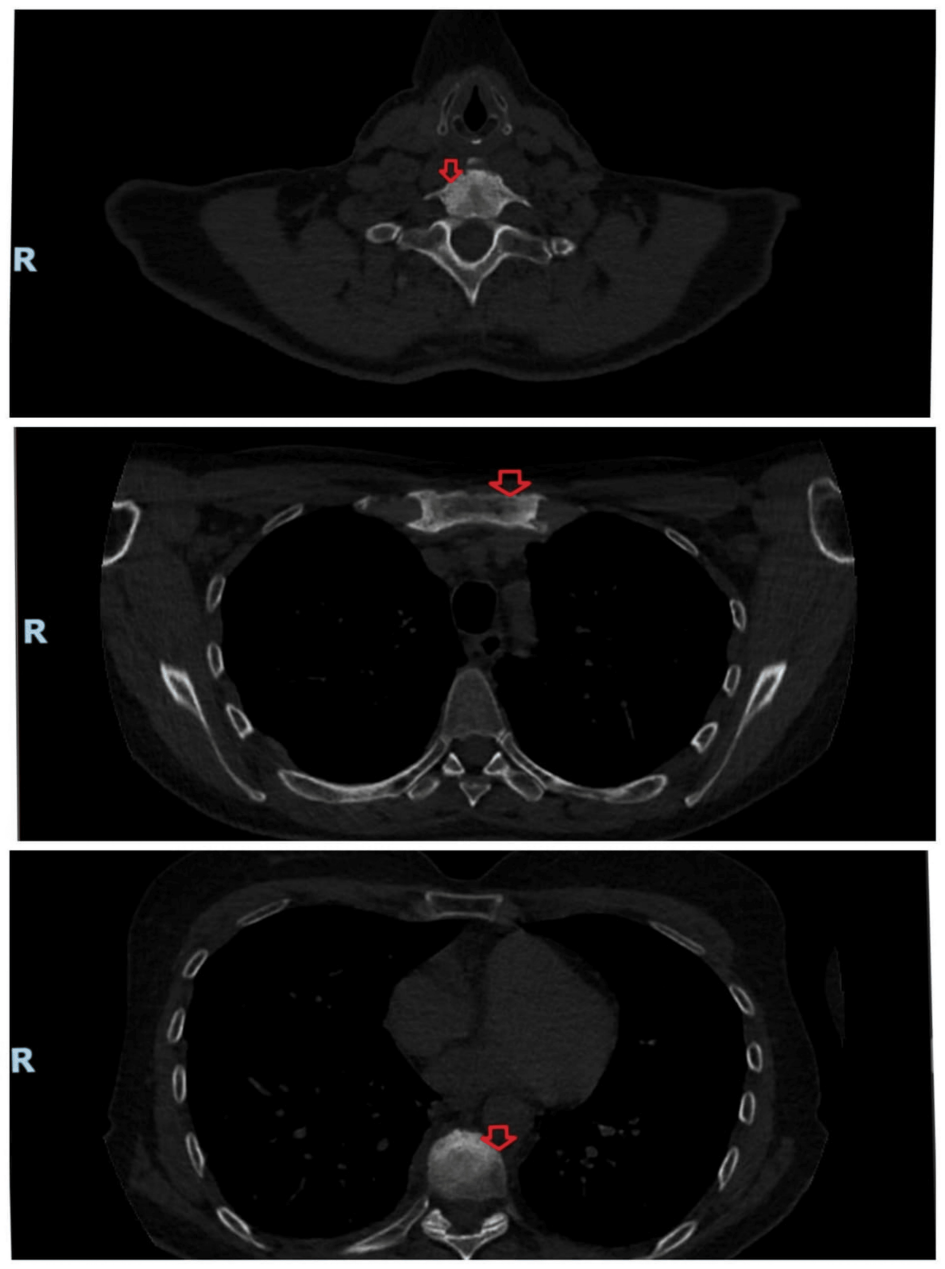
Follow-up axial CT Scan bone window shows subtle improvement in the appearance of the sclerosis within the sternum, lower cervical, and mid-thoracic spine.

During the follow-up visits, reductions in shoulder and rib pain and improvements in night sweats and mobility were noted. A repeat MRI scan and CT scan done six months later were consistent with the features of SAPHO syndrome, with subtle improvement (some healing in the depression of the superior end) after treatment (Figure 3-4).

In this case, the patient initially presented to primary care with symptoms suggestive of recurrent chest infections. However, a subsequent CT scan raised suspicions of bony metastases, prompting extensive investigations for a primary malignancy, which were ultimately inconclusive. Eventually, the correct diagnosis of SAPHO syndrome was made, as the patient's clinical presentation and imaging did not align with any other conditions considered.

## Discussion

SAPHO syndrome, characterized by a constellation of musculoskeletal and cutaneous manifestations, presents a unique challenge in diagnosis and management. Though the etiology of the said disease is unknown, it is believed that genetic (most frequently associated with HLA-B27), infectious, immunological, and environmental factors play a role in the pathogenesis [5].

Symptoms vary among individuals and usually include osteoarticular involvement (osteitis, hyperostosis, synovitis), causes chronic pain and swelling of the bones and joints, especially anterior chest wall/ spine/ pelvis, Cutaneous involvement includes palmoplantar pustulosis and severe acne (can manifest as acne conglobata, acne fulminans or hidradenitis suppurativa), Fever and fatigue. Plain radiographs may reveal osteitis and hyperostosis of the sternoclavicular joint, sacroiliitis, and osteosclerosis of vertebral bodies [6]. The diagnostic value of laboratory testing is limited, with only 13-30% of SAPHO patients testing positive for the human leukocyte antigen (HLA) B27 [7]. Bone scintigraphy shows increased uptake (Bull's head sign) and helps in revealing asymptomatic/early bone involvement or abnormalities not detectable on radiographs [3,5]. 

The current case report illustrates an atypical presentation of SAPHO syndrome, where the patient exhibited only a subset of the common symptoms (osteoarticular), leading to diagnostic delays. This presentation aligns with existing literature, highlighting the syndrome's heterogeneous nature [8]. For instance, a study by Kahn et al. (1994) [9] emphasized that the clinical manifestations of SAPHO syndrome can differ significantly among patients, with some presenting predominantly with osteoarticular symptoms while others exhibit severe dermatological involvement. This variability complicates the diagnostic process, as illustrated in our case, where initial assessments focused more on ruling out malignancy rather than considering SAPHO syndrome.

In terms of diagnostic imaging, our case involved a comprehensive workup, including radiographs, CT and MRI. MRI is highlighted as a critical tool for identifying active inflammatory lesions in SAPHO syndrome, which can often be missed on plain radiographs. In our case, while MRI provided valuable insights into the extent of the sclerosis, the initial reliance on radiographs may have contributed to a slightly delayed diagnosis.

Therapy is empirical and aimed at relieving pain and modifying the inflammatory process. It includes non-steroidal anti-inflammatory drugs (NSAIDs), anti-rheumatic drugs, corticosteroids, antibiotics, bisphosphonates, and disease-modifying agents. Dermatological management includes topical corticosteroids, antimicrobial agents, psoralen-ultraviolet A (PUVA), and retinoids [4]. 

Therapeutic strategies in the current case included a combination of NSAIDs and corticosteroids, which are consistent with the empirical treatment approaches. 

In short, this case report highlights the complexities of diagnosing SAPHO syndrome and emphasizes the importance of considering the full spectrum of clinical manifestations and utilizing comprehensive diagnostic approaches. The similarities and differences observed in our case compared to existing literature underscore the need for heightened awareness among clinicians regarding this rare syndrome. By recognizing the potential for atypical presentations and the limitations of current diagnostic tools, we can improve the timeliness of diagnosis and optimize management strategies for patients with SAPHO syndrome.

The strengths of this case report include a thorough, comprehensive diagnostic workup and a multidisciplinary approach. The limitations are delayed diagnosis of SAPHO syndrome, primarily due to the initial diagnostic focus on malignancy and the late recognition of the associated skin manifestations. This delay in identifying the dermatological features contributed to a protracted diagnostic process.

## Conclusions

To conclude, this case involves a woman with progressive musculoskeletal pain who was ultimately diagnosed with SAPHO syndrome following extensive investigations to exclude malignancy had taken place. The diagnostic process was challenging due to the absence of typical skin manifestations. The diagnosis was confirmed after multiple scans and a multidisciplinary team meeting. Treatment with prednisolone and azathioprine resulted in modest symptom improvement. Immunosuppressive therapies, including corticosteroids and disease-modifying anti-rheumatic drugs (DMARDs), may provide symptomatic relief in such cases, but we don't have set guidelines for treating the disease.

This case underscores the importance of considering SAPHO syndrome in patients presenting with unexplained bone pain, even in the absence of characteristic skin lesions such as acne or pustulosis and when imaging reveals sclerotic lesions in areas like the sternum, clavicle, or spine. Further data collection and research are necessary to establish comprehensive treatment guidelines.
